# Molecular epidemiology and virulence factors of group B *Streptococcus* in South Korea according to the invasiveness

**DOI:** 10.1186/s12879-024-09625-1

**Published:** 2024-07-26

**Authors:** Jae Hong Choi, Tae Hyoung Kim, Eui Tae Kim, Young Ree Kim, Hyunju Lee

**Affiliations:** 1 Pediatrics, Jeju National University College of Medicine, Jeju, Republic of Korea; 2 Pediatrics, Jeju National University Hospital, Jeju, Republic of Korea; 3 Biomedicine and Drug Development, Jeju National University College of Medicine, Jeju, Republic of Korea; 4 Microbiology and Immunology, Jeju National University College of Medicine, Jeju, Republic of Korea; 5 Laboratory Medicine, Jeju National University College of Medicine, Jeju, Republic of Korea; 6 Pediatrics, Seoul National University Bundang Hospital, Seongnam, Republic of Korea; 7 Pediatrics, Seoul National University College of Medicine, Seoul, Republic of Korea

**Keywords:** *Streptococcus agalactiae*, Group B *Streptococcus*, Virulence factor, Multilocus sequence typing

## Abstract

**Background:**

Group B *Streptococcus* (GBS) causes invasive infections in newborns and elderly individuals, but is a noninvasive commensal bacterium in most immunocompetent people. Recently, the incidence of invasive GBS infections has increased worldwide, and there is growing interest in the molecular genetic characteristics of invasive GBS strains. Vaccines against GBS are expected in the near future. Here, we aimed to analyze the molecular epidemiology of GBS according to the invasiveness in South Korea.

**Methods:**

We analyzed GBS isolates collected and stored in two hospitals in South Korea between January 2015 and December 2020. The invasiveness of these isolates was determined via a retrospective review of clinical episodes. Totally, 120 GBS isolates from 55 children and 65 adults were analyzed. Serotype and sequence type (ST) were determined using multiplex polymerase chain reaction (PCR) and multilocus sequence typing, respectively. Fourteen virulence factor-encoding genes of GBS were analyzed using multiplex PCR.

**Results:**

Forty one (34.2%) were invasive infection-related GBS isolates (iGBS). The most frequently detected serotype was III (39/120, 32.5%), and it accounted for a high proportion of iGBS (21/41, 51.2%). The most frequent ST was ST19 (18/120, 15.0%), followed by ST2 (17/120, 14.2%). Serotype III/ST17 was predominant in iGBS (12/41, 29.3%), and all 17 ST2 strains were noninvasive. The distribution of most of the investigated virulence factors was not significantly related to invasiveness; noteworthily, most of the serotype III/ST17 iGBS carried pilus island (PI) 2b (10/12, 83.3%), and the prevalence of *fbsB* was significantly low compared with noninvasive GBS isolates (*P* = 0.004). Characteristically, the combination of *bca*(+)-*cspA*(+)-*pavA*(+)-*fbsB*(-)-*rib*(+)-*bac*(-) was predominant in iGBS (24.4%, 10/41).

**Conclusions:**

Serotype III/ST17 GBS carrying PI-2b was frequently detected in iGBS. There was no significant association between invasiveness and the pattern of virulence factors; however, a specific combination of virulence factors was predominant in iGBS.

## Background

*Streptococcus agalactiae* (group B *streptococcus*, GBS) is the leading cause of invasive infections in neonates [1] and opportunistic infections in adults [2]. Invasive diseases associated with GBS infection include bacteremia, meningitis, endocarditis, osteoarticular infections, and pneumonia. In South Korea, GBS is the most common bacterial pathogen causing invasive infections in infants aged less than 3 months [3]. In addition to invasive infections, GBS can colonize the genitourinary and gastrointestinal tracts of healthy adults and pregnant women under non-invasive conditions [4, 5].

The estimated global incidence of neonatal GBS infection including GBS associated preterm birth and stillbirth is 0.49 per 1000 individuals [6]. Recently, the incidence of GBS infections has increased in older adults worldwide [7, 8]. Furthermore, a marked increase in the incidence of GBS infections has been reported in elderly patients with diabetes mellitus and cancer, and those undergoing renal dialysis [9]. The reason of the increase in these cases in not fully understood. Some experts suggested that this increase may be associated with the aging population or changing characteristics of GBS such as serotypes [10, 11]. Several preventive efforts such as GBS screening in pregnancy and intrapartum antibiotic prophylaxis have been adopted. These strategies have markedly decreased early-onset GBS (EOGBS) infections in neonates; however, they have negligible effect on late-onset GBS (LOGBS) infections. In this context, administration of vaccines against GBS is considered an effective preventive method. Although vaccines against GBS have not yet been commercialized, several polysaccharide capsule-based vaccines for pregnant women are expected in the near future [12]. Classically, GBS was classified into 10 serotypes by surface capsular polysaccharides, and that have been studied for the main target of GBS vaccines. Molecular genetic research on GBS has proposed various virulence factors such as pilus island (PI) alleles and surface alpha-like proteins as targets of GBS vaccines [13].

Considering the gradually increasing trend of invasive infections and the development of vaccines against GBS, it is important to monitor the changes in molecular genetic characteristics of GBS. In this study, we aimed to analyze the molecular epidemiology of GBS in South Korea according to the invasiveness, focusing on virulence factors.

## Methods

### Study population and specimens

From January 2015 to December 2020, a total of 120 GBS isolates were analyzed. The isolates were collected from Seoul National University Bundang Hospital (30 isolates) and Jeju National University Hospital (90 isolates) in Korea and stored at -70 °C via a hospital-wide surveillance system. The samples were obtained from 55 (45.8%) children and 65 (54.2%) adults. GBS strains were isolated from blood (*n* = 29), cerebrospinal fluid (*n* = 11), tracheal aspirate (*n* = 8), urine (*n* = 43), vaginal discharge (*n* = 24), and wound samples (*n* = 4). We determined the invasiveness of GBS through a retrospective medical chart review. Invasive infection-related GBS isolates (iGBS) were identified based on sterile isolation sites and proper invasive clinical episodes.

### Analysis of GBS isolates

GBS isolates were identified, and antimicrobial susceptibility tests were performed using an automated microbiology system, Vitek II ID-GPC (bioMérieux, Durham, NC, USA). Automated susceptibility test results were obtained for penicillin, clindamycin, erythromycin, and ciprofloxacin. DNA from GBS isolates was purified using the Solg™ Genomic DNA Prep Kit (Solgent, Daejeon, South Korea), according to the manufacturer’s protocol. GBS serotypes were determined using modified multiplex PCR assays based on the analysis of unique band patterns [14]. Total 20µL PCR mixture using 1.6µL of template DNA with primers [14]. The samples were amplified by a denaturation step for 5 min at 95℃, followed by 25 cycles of 95℃ for 30s, 53℃ for 30s, and 72 for 1 min and a final cycle of 72℃ for 10 min. The PCR products were analyzed by electrophoresis in 1.5% agarose gel. Multilocus sequence typing (MLST) was performed by sequencing seven housekeeping genes (*adh*, *pheS*, *atr*, *glnA*, *sdhA*, *glcK*, and *tkt*) using Sanger sequencing. Sequencing reactions were performed in the DNA Engine Tetrad 2 Peltier Thermal Cycler (Bio-Rad, Hercules, CA, USA) using the ABI BigDye^®^ Terminator v3.1 Cycle Sequencing kit (Applied Biosystems, Waltham, MA, USA) in Macrogen Corporation (Seoul, Korea). Specific primers for these genes were available in *Streptococcus agalactiae* MLST database (https://pubmlst.org/sagalactiae). And number of matched alleles of each of the seven housekeeping genes was used to determine the sequence type (ST). The STs were clustered into a clonal complex (CC) using the goeBURST PHYLOViZ program (https://phyloviz.readthedocs.io/en/latest/index.html), and phylogenetic relationships were represented in a diagram according to the invasiveness at single locus variant levels.

After screening researches focusing virulence factor-encoding genes of GBS, the following 14 genes were selected and analyzed: *fbsA*, *fbsB*, *psvA*, *lmp*, *scpB*, *bac*, *bca*, *cfb*, *cspA*, *cylE*, *hylB*, *rib*, *pbp1A/ponA*, and pilus-related genes. We divided these genes into four sets for multiplex PCR according to base pair size (Table 1). The amplification conditions were similar to those of a previous study [15], with some modifications such as changes of reaction cycles or temperature. A reaction mixture of total volume 20 µL was prepared by mixing 10 µL of 2× Taq Master Mix, 1 µL of template DNA, 0.5 µL of each primer, and 8 µL of distilled water. Initial denaturation at 95 °C for 5 min was followed by 38 cycles of amplification at 94 °C for 30 s, annealing at speci fic temperatures (47 °C for set A, 45 °C for set B, 47 °C for set C, and 52 °C for set D) for 30 s, and a final extension step at 72 °C for 30 s (1 min for set D). The amplified PCR products were visualized on 1.5% agarose gels. The phylogenetic tree was represented using UPGMA algorithm and the distance was expressed as Hamming distance. This tree was combined with the invasiveness and the combination of 6 virulence factors which were detected less than 95.0% of GBS isolates.

**Table 1:** Primers used to amplify virulence factor-encoding genes of GBS

Virulence factor-encoding gene	Sequence (5′ to 3′)	Amplicon size (bp)
Set A		
*cspA*	F: CTGCTAAAGCACACCTAAAC, R: ATCAGTAGTGGTTCCTTTCC	971
*pavA*	F: TACTACCAAGAGAAGGCTGA, R: GGAGAGACGAGCTTTAGAGT	729
*cylE*	F: GTACATTAGGTGCCTTTGG, R: TACTCAGCCTTTCTCCATC	564
*hylB*	F: CTATGCTGACGGTTCTTAC, R: AGGTCTAAGTTTCGCTCTT	323
*lmb*	F: TCAGTTAGTTGCTCTGCTTC, R: CTTTATGACCCACATACCTG	152
Set B		
*fbsB*	F: CACTCGATAACACTGTGGAT, R: CTGGAACTGTTTCTGTCTTG	936
*scpB*	F: ACAACGGAAGGCGCTACTGTTC, R: ACCTGGTGTTTGACCTGAACTA	255
*bca*	F: TAACAGTTATGATACTTCACAGAC, R: ACGACTTTCTTCCGTCCACTTAGG	535
Set C		
*pbp1A/ponA*	F: AGGGGTAGTAGCATTACCAT, R: CAACTATATGACTGGGATCG	939
*bac*	F: CTCCAAGCTCTCACTCATAG, R: GAAACATCTGCCACTGATAC	750
*cfb*	F: GGATTCAACTGAACTCCAAC, R: GACAACTCCACAAGTGGTAA	600
*rib*	F: GGGGTTACACAAGGTAATCT, R: TCCACTTAGGATCGTTTG	425
*fbsA*	F: AACCGCAGCGACTTGTTA, R: AAACAAGAGCCAAGTAGGTC	278
Set D (pilus site)		
PI-1	F: GGTCGTCGATGCTCTGGATTC, R: GTTGCCCAGTAACAGCTTCTCC	881
PI-2a	F: CTATGACACTAATGGTAGAAC, R: CACCTGCAATAGACATCATAG	575
PI-2b	F: ACACGACTATGCCTCCTCATG, R: TCTCCTACTGGAATAATGACAG	721

GBS group B *Streptococcus*, PI pilus island

### Statistical analyses

All data analyses were performed using software R version 3.6.2. (R Foundation for Statistical Computing, Vienna, Austria). Categorical variables are expressed as percentage; they were analyzed using the Chi-square test. Continuous variables are expressed as mean ± standard deviation. Results with *P* < 0.05 were considered statistically significant.

## Results

In this study, the mean age (± standard deviation) of the participants who had GBS isolates was 31.12 ± 32.24 years. Most GBS isolates were obtained from children, pregnant women in their 30s, and elderly individuals in their 80s (Table 2). Among the 120 GBS isolates, 41 (34.2%) were classified to be iGBS, which mostly infected children (37/41, 90.2%). The mean age of the 37 children who had iGBS infection was 0.25 ± 0.72 years, and 5 (13.5%) of them were categorized as having EOGBS infections.

**Table 2:** Age distribution of patients in whom GBS was detected according to invasiveness

Age (years)	Total *N* (%)	Invasive infection *N* (%)	Noninvasive infection *N* (%)
0–0.3	45 (37.5)	33 (80.5)	12 (15.2)
0.3–9	9 (7.5)	4 (9.8)	5 (6.3)
10–19	1 (0.8)	0	1 (1.3)
20–29	1 (0.8)	0	1 (1.3)
30–39	24 (20.0)	0	24 (30.4)
40–49	9 (7.5)	0	9 (11.4)
50–59	4 (3.3)	0	4 (5.1)
60–69	3 (2.5)	1 (2.4)	2 (2.5)
70–79	9 (7.5)	1 (2.4)	8 (10.1)
80–89	12 (10.0)	1 (2.4)	11 (13.9)
90–99	3 (2.5)	1 (2.4)	2 (2.5)
Total	120	41	79

GBS group B *Streptococcus*

All 120 GBS isolates were susceptible to penicillin. The resistance rates were 45.0% (54/120) for clindamycin, 49.6% (58/117) for erythromycin, and 29.3% (34/116) for ciprofloxacin, relatively. The difference in antibiotic susceptibility according to invasiveness was not significant. Among all isolates, the most frequent capsular serotypes were III (39/120, 32.5%), V (20/120, 16.7%), and VIII (20/120, 16.7%) (Table 3). Serotype III accounted for the highest proportion of iGBS (21/41, 51.2%), and the majority of GBS strains causing meningitis were serotype III (8/11, 72.7%). The proportions of serotypes Ia and IV were as low as 5.8% (7/120) and 3.3% (4/120), respectively. However, these two serotypes mainly caused invasive infections, with serotype Ia accounting for 71.4% (5/7) and serotype IV for 100% (4/4). All of the 20 serotype VIII isolates were noninvasive.

**Table 3:** GBS serotypes according to invasiveness and isolated specimens

Serotype	Total isolates	Invasive infection				Noninvasive infection	
		*N* (%)		Specimen (*N*)	*N* (%)		Specimen (*N*)
Ia	7 (5.8)	5 (12.2)	B (4), C (1)		2 (2.5)		Urine (2)
Ib	10 (8.3)	2 (4.9)	B (2)		8 (10.1)		T (1), U (3), V (2), W (2)
II	6 (5.0)	0 (0.0)			6 (7.6)		T (1), U (2), V (3)
III	39 (32.5)	21 (51.2)	B (13), C (8)		18 (22.8)		U (10), V (7), W (1)
IV	4 (3.3)	4 (9.8)	B (3), J (1)		0 (0.0)		
V	20 (16.7)	5 (12.2)	B (4), C (1)		15 (19.0)		U (13), V (2)
VI	9 (7.5)	3 (7.3)	B (2), C (1)		6 (7.6)		T (2), U (2), V (2)
VIII	20 (16.7)	0 (0.0)			20 (25.3)		T (3), U (8), V (8), W (1)
NT	5	1			4		
Total	120	41			79		

N, number; NT, nontypable serotype; B, blood; C, cerebrospinal fluid; J, joint aspirates; T, tracheal aspirates; U, urine; V, vaginal fluid; W, wound

.

**Fig. 1:**
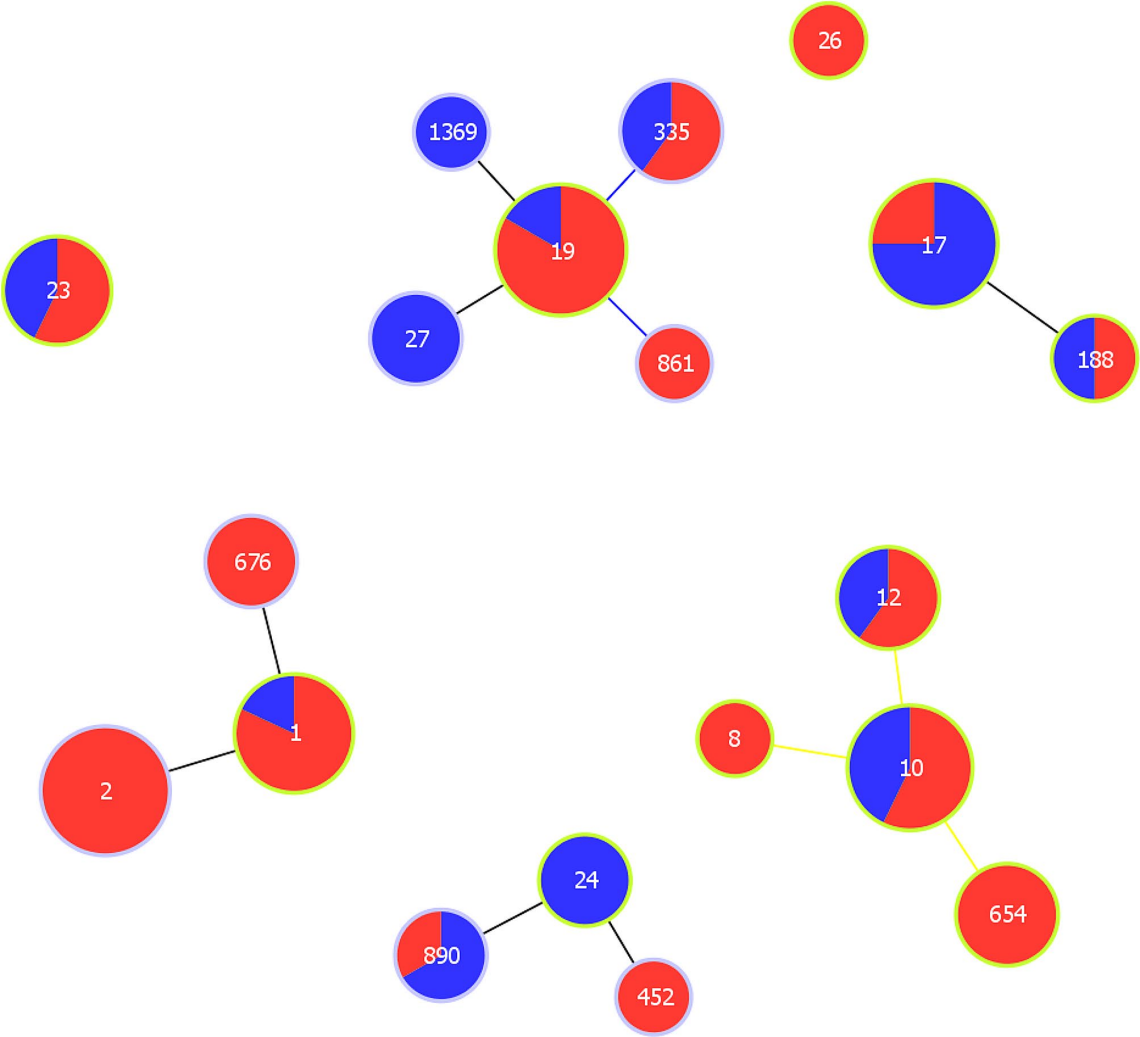
Phylogenetic diagram of GBS isolates according to invasiveness. Phylogenetic relationships were identified using goeBURST analyses at single locus variant levels. Numbers represent sequence types (STs) based on the multilocus sequence typing (MLST) analysis. Invasiveness is represented by color: red, noninvasive GBS isolates; blue, invasive GBS isolates

In total, 19 STs were identified and grouped into seven CCs. Distributions of STs according to serotypes were shown in Table 4. The most common STs were ST19 (18/120, 15.0%), ST2 (17/120, 14.2%), ST17 (16/120, 13.3%), and ST10 (14/120, 11.7%). The phylogenetic relationships and the proportion of iGBS of each ST are illustrated in Fig. 1. All ST2 (*n* = 17) isolates were noninvasive and most ST19 isolates (15/18, 83.3%) were noninvasive. Among iGBS, ST17 was the most common (12/41, 29.3%), followed by ST10 (6/18, 14.6%). All ST17 (*n* = 12) iGBS strains were serotype III and all ST24 (*n* = 3) iGBS strains were serotype Ia (Table 5).

**Table 4:** Association between serotypes and sequence types

CC/ST	*N* (%)	Serotype								
		Ia	Ib	II	III	IV	V	VI	VIII	NT
CC1	31(25.8)	0	0	2	2	0	4	3	18	2
ST1	11	0	0	1	2	0	4	3	1	0
ST2	17	0	0	0	0	0	0	0	15	2
ST676	3	0	0	1	0	0	0	0	2	0
CC12	25(20.8)	0	9	2	3	0	3	5	1	2
ST8	1	0	0	1	0	0	0	0	0	0
ST10	14	0	3	0	0	2	2	5	1	1
ST12	5	0	2	1	0	1	1	0	0	0
ST654	5	0	4	0	0	0	0	0	0	1
CC17	18(15.0)	0	0	0	18	0	0	0	0	0
ST19	16	0	0	0	16	0	0	0	0	0
ST27	2	0	0	0	2	0	0	0	0	0
CC19	28(23.3)	0	0	0	18	0	8	1	0	1
ST19	18	0	0	0	10	0	7	0	0	1
ST27	3	0	0	0	2	0	0	1	0	0
ST335	5	0	0	0	5	0	0	0	0	0
ST861	1	0	0	0	1	0	0	0	0	0
ST1369	1	0	0	0	0	0	1	0	0	0
CC23 (ST23)	7(5.8)	2	1	2	0	0	3	0	0	0
CC24	7(5.8)	5	0	1	0	0	1	0	0	0
ST24	3	3	0	0	0	0	0	0	0	0
ST452	1	1	0	0	0	0	0	0	0	0
ST890	3	1	0	1	0	0	1	0	0	0
CC26 (ST26)	1(0.8)	0	0	0	0	0	1	0	0	0

CC, clonal complex; ST, sequence type; NT, nontypable

**Table 5:** Sequence types and serotypes of invasive GBS isolates

Sequence type (ST)	Number (%)	Serotypes (number)
ST1	2 (4.9)	III (1), V (1)
ST10	6 (14.6)	IV (2), V (1), VI (2), NT (1)
ST12	2 (4.9)	Ib (1), IV (1)
ST17	12 (29.3)	III (12)
ST19	3 (7.3)	III (3)
ST23	3 (7.3)	Ia (1), Ib (1), V (1)
ST24	3 (7.3)	Ia (3)
ST27	3 (7.3)	III (2), VI (1)
ST188	1 (2.4)	III (1)
ST335	2 (4.9)	III (2)
ST890	2 (4.9)	Ia (1), V (1)
ST1369	1 (2.4)	V (1)
New	1 (2.4)	IV (1)

GBS group B *Streptococcus*

The four patterns of PIs and their distribution according to serotype and invasiveness are shown in Table 6. All 120 GBS isolates harbored either PI-2a (70.8%, 85/120) or PI-2b (29.2%, 35/120), and PI-1 was detected in 58.3% of isolates (70/120). The combination of PI-1 and PI-2a was the most frequently detected (53/120, 44.2%). The GBS isolates carrying PI-1 (regardless of the presence of PI-2) were more prevalent in the nGBS group (*P* = 0.034). Among iGBS carrying PI-2b (regardless of PI-1 status), 90.9% (10/11) were serotype III/ST17. A combination of PI-1 and PI-2b was detected in nGBS (88.2%, 15/17) and serotype VIII (82.4%, 14/17). The pattern of PIs was not related to the isolated sites.

**Table 6:** Distribution of pilus island according to the invasiveness and serotypes

	PI-1/ PI-2a	PI-1/ PI-2b	PI-2a	PI-2b
iGBS	16 (30.2%)	2 (11.8%)	14 (43.8%)	9 (50%)
Blood (29)	13	2	8	6
CSF (11)	3	0	5	3
nGBS	37 (69.8%)	15 (88.2%)	18 (56.2%)	9 (50%)
Vagina (24)	10	6	5	3
Urine (43)	21	6	11	5
Trachea (8)	4	2	1	1
Wound (4)	2	1	1	0
Serotype				
Ia	1	0	5	1
Ib	5	0	4	1
II	3	0	3	0
III	20	2	4	13
IV	1	0	2	1
V	13	0	7	0
VI	5	0	4	0
VIII	2	14	2	2
NT	3	1	1	0
Total	53	17	32	18

PI, pilus island; iGBS, invasive GBS isolates; nGBS, noninvasive GBS isolates; CSF, cerebral spinal fluid; NT, nontypable serotype

**Fig. 2:**
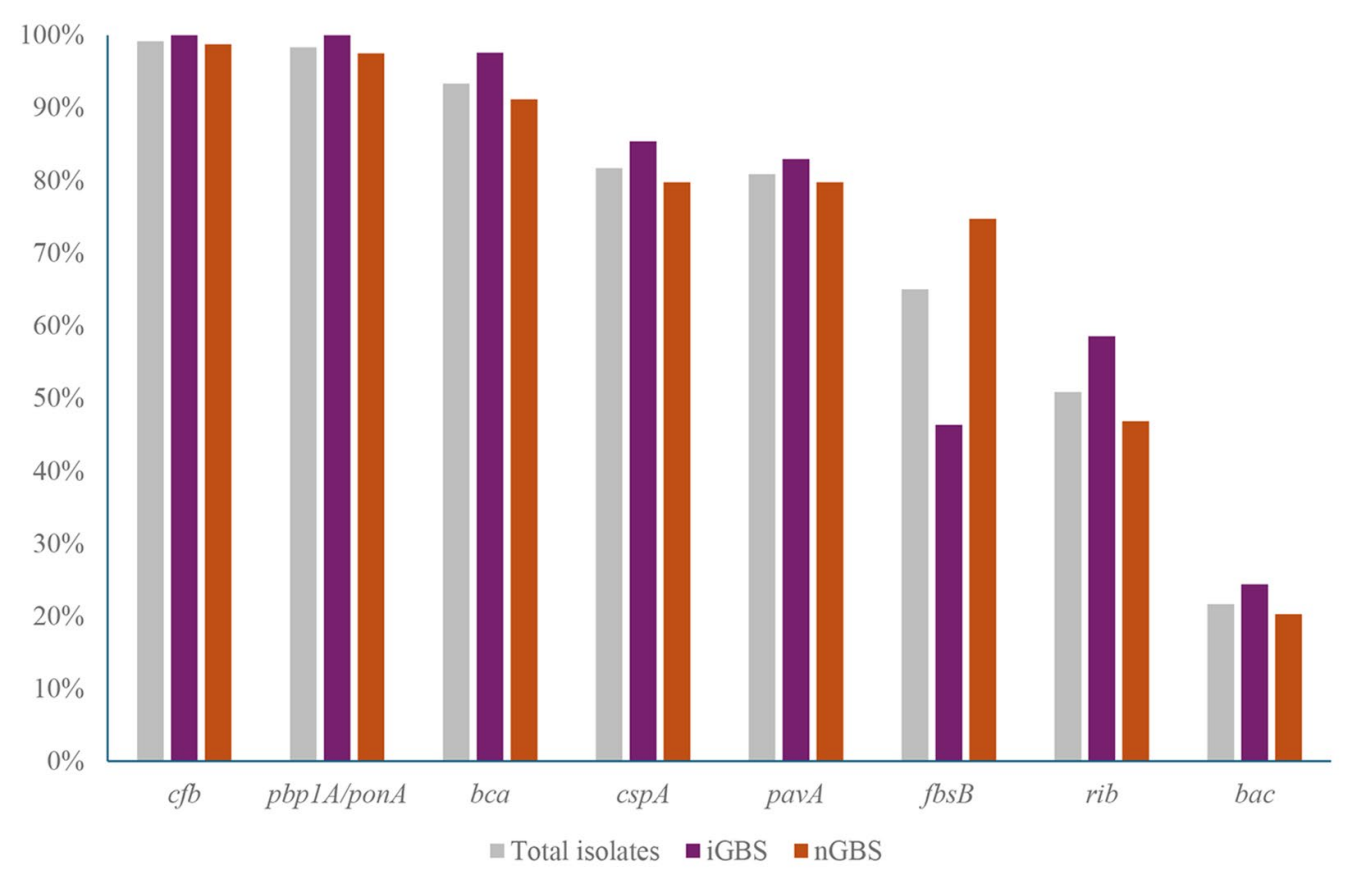
Proportion of virulence factor-encoding genes according to invasiveness. Five virulence factor-encoding genes found in all GBS isolates were excluded. iGBS, invasive GBS isolates; nGBS, noninvasive GBS isolates. *P < 0.005

**Fig. 3:**
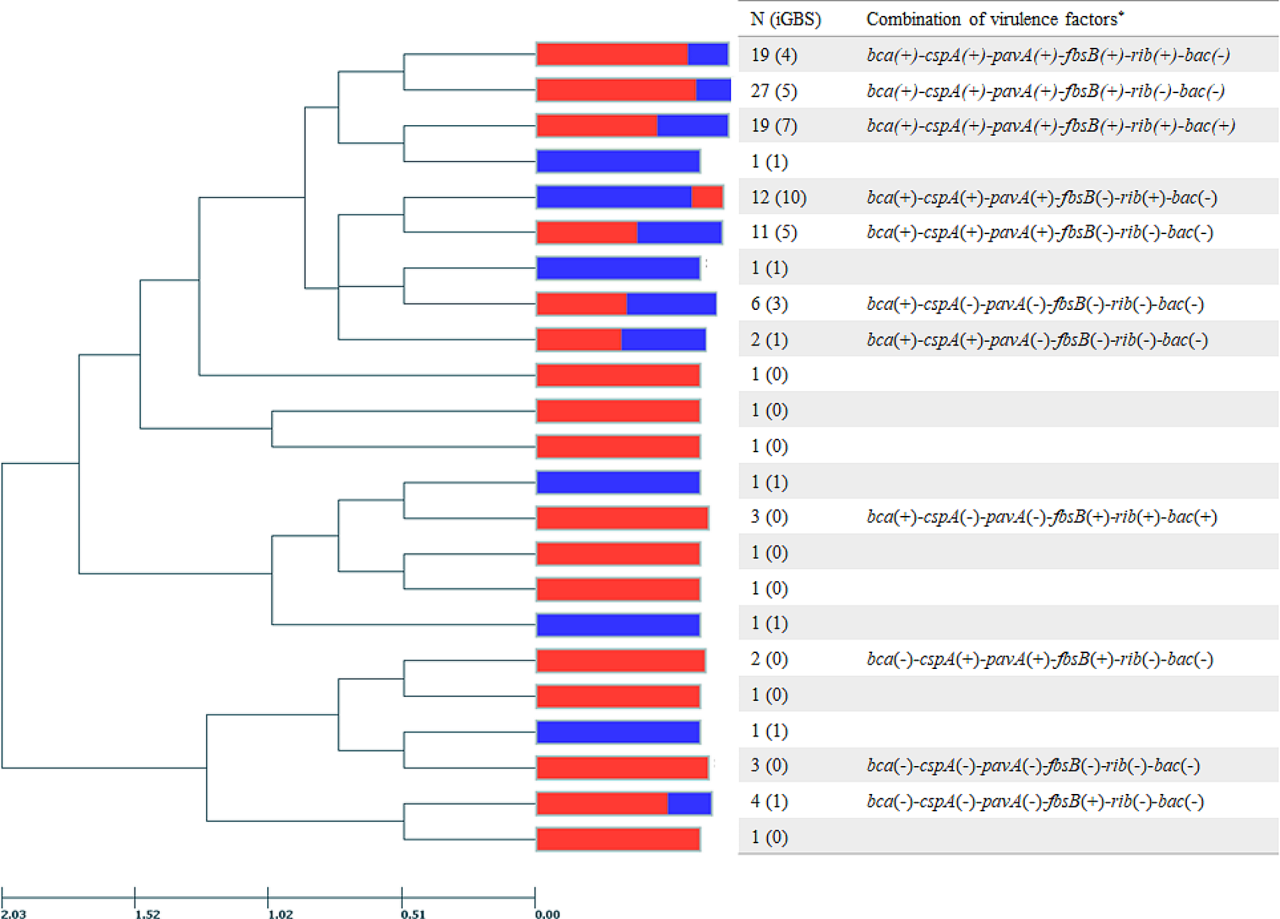
Phylogenetic tree of GBS isolates according to the combination of virulence factors. Among 13 virulence factors, 6 virulence factors whose detection rate was less than 95.0% were selected to represent only the significant factors. Combinations of virulence factors with two or fewer isolates were omitted and represented as blank columns. The tree was constructed using the UPGMA algorithm and the distance is expressed as Hamming distance. The red area indicates the proportion of nGBS and the blue area indicates that of iGBS. UPGMA, unweighted pair group method with arithmetic mean; iGBS, invasive GBS isolates; nGBS, noninvasive GBS isolates

Five virulence factor-encoding genes (*cylE*, *hylB*, *lmb*, *scpB*, and *fbsA*) were detected in all 120 GBS isolates. The distribution of other virulence factor-encoding genes was as follows: *cfb* (119/120, 99.2%), *pbp1A/ponA* (118/120, 98.3%), *bca* (112/120, 93.3%), *cspA* (98/120, 81.7%), *pavA* (97/120, 80.8%), *fbsB* (78/120, 65.0%), *rib* (61/120, 50.8%), and *bac* (26/120, 21.7%) (Fig. 2). The prevalence of *fbsB* was significantly lower (*P* = 0.004) in iGBS compared with nGBS. And the prevalence of *rib* was relatively high in iGBS (58.5% compared with 46.8% in nGBS), however the difference was not significant (*P* = 0.306). The following combination of six virulence factors, excluding those commonly present in both iGBS and nGBS, was predominant in iGBS (10/41): the *bca*(+)-*cspA*(+)-*pavA*(+)-*fbsB*(-)-*rib*(+)-*bac*(-) (Fig. 3). Characteristically, all three ST24 isolates caused invasive infections and had the same virulence factor patterns as described above.

## Discussion

This study revealed several specific molecular characteristics of iGBS in South Korea. Among iGBS, serotype III was the most common (51.2%), and all of the 20 serotype VIII isolates were nGBS. Among the three prevalent STs (ST19, ST2, and ST17), most ST19 (83.3%) and ST2 (100%) isolates were nGBS. Serotype III/ST17 was the main iGBS strain, and it was found to have PI-2b regardless of PI-1 status (10/12, 83.3%). There were no significant differences of the distribution of the analyzed virulence factor-encoding genes according to invasiveness; however, a specific combination of virulence factor-encoding genes was predominant in iGBS.

Maternal GBS colonization is a major determinant of EOGBS infections in neonates. European studies have shown similarity in serotype distribution between maternal colonized GBS and iGBS, and this finding indicates that EOGBS infection is caused by GBS transmission from mother to newborn [16, 17]. In this study, serotype VIII (8/24, 33.3%) and serotype III (7/24, 29.2%) were the main serotypes of GBS isolated from vaginal discharge. The recent serologic data of GBS among pregnant South Korean women showed that the most common serotypes were V (22.7%) and VIII (20.0%) [18, 19]. However, globally, the frequently distributed serotypes of maternal colonized GBS are I (Ia/Ib), III, and V [5]. In South Korea, the incidence of LOGBS was relatively high compared to the global average [20, 21]. A similar trend of a higher incidence of LOGBS infection was observed in the present study (LOGBS/EOGBS = 5.4). The relatively low prevalence of EOGBS infection in South Korea may be attributed to the high incidence of serotype VIII in isolates from vaginal discharge, which is not highly associated with iGBS infection. This epidemiology is not fully understood, and further study for serotype VIII GBS is needed to be verified.

In this study, the distribution of serotypes and STs between the iGBS and nGBS groups differed significantly. The serotype III/ST17 strain was the most prevalent in iGBS, whereas the serotype VIII/ST2 strain was the most common in nGBS. These results are similar to the distribution of iGBS serotypes with predominant serotype III followed by Ia, Ib, II, and V in other countries [16, 22].

Pili are cell-wall-anchored appendages on GBS cell surface; they play an important role in bacterial attachment to epithelial cells. Three pilus types have been identified in GBS, and all GBS strains have the at least one PI variant [23]. The most frequently detected PI combination is PI-1 and PI-2a [24–26], consistent with the results of this study. Hypervirulent ST17 GBS isolates are known to contain the combination of PI-1 and PI-2b, and this combination is rare among other STs [27]. The role of PI-2b in mediating the interaction between GBS and host cell is well known, especially in ST17 GBS [28]. We found a distinctive pattern where PI-2b without PI-1 was dominant in serotype III iGBS. Additionally, several PI-1 and PI-2b combinations were identified in serotype VIII nGBS (Table 6).

The laminin-binding protein encoded by *lmb* mediates the attachment of GBS to human laminin, and this attachment is crucial for colonization [29]. Fibrinogen-binding protein A (encoded by *fbsA*) and C5a peptidase (encoded by *scpB*) help bacteria bind to epithelial cells and fibronectin, respectively. Almost all GBS isolates from humans harbor these virulence factors, which were detected in all GBS isolates in this study. Fibrinogen-binding protein B (*fbsB*), along with *fbsA*, was detected in CC17 GBS strains, and the high fibrinogen-binding ability may contribute the invasiveness of GBS in neonates [30]. However, *fbsB* was rather less frequently detected in iGBS (Fig. 2) and *fbsB* was mostly detected in non-CC17 strains in this study. Although research on various components of GBS is limited, rib proteins (encoded by *rib*) and C proteins (encoded by *bac* and *bca*) have been considered as vaccine candidates, in addition to capsular polysaccharides and pili [31, 32]. This study revealed differences in the prevalence of these three virulence factors between the iGBS and nGBS groups, with various combinations exhibiting no specific trends. However, the combination of *bca*(+)-*cspA*(+)-*pavA*(+)-*fbsB*(-)-*rib*(+)-*bac*(-) was predominant in the iGBS group. Notably, all ST24 serotype Ia iGBS strains exhibited identical virulence patterns. A Spanish study from the early 2000s reported that the ST24/*bca* sublineage of serotype Ia might emerge as an important cause of neonatal invasive infections, despite its limited prevalence [33]. This strain may have maintained consistent genotypic pattern unaffected by geographical and temporal variations.

This study had several limitations. Despite collecting isolates over five years from the two hospitals, the number of GBS was relatively small. The hospital surveillance systems were passive surveillance systems, and there were fewer isolates in the early stage, leading to a lack of consistency. Furthermore, the majority of the invasive strains were from children, and the number of iGBS in adults was limited. GBS strains have multiple virulence factors, and not all were included in the study. However, analyzing the molecular characteristics of GBS through a comprehensive comparison between iGBS and nGBS offers valuable insights, and to the best of our knowledge, this is the first study to focus on pili and various virulence factors of GBS in South Korea.

## Conclusions

The distribution of serotypes and STs according to invasiveness determined in the present study was similar to that in previous research. The distribution of the PI-1 and PI-2b combination according to serotype was unique, and a specific combination of virulence factors was found. The technical development of several capsular polysaccharide-based GBS vaccines has been completed, and GBS vaccines may soon be commercialized in South Korea. In this milieu, monitoring GBS molecular characteristics in various populations and isolates before and after vaccine implementation is crucial.

## Data Availability

The dataset used in this study are original. The dataset analyzed during the current study are available from corresponding author on reasonable request.

## Abbreviations

CSF: Cerebral spinal fluid
CC: Clonal complex
EOGBS: Early-onset group B *Streptococcus*
GBS: Group B infection-related *Streptococcus*
iGBS: Invasive group B *Streptococcus* isolate
LOGBS: Late-onset group B *Streptococcus*
MLST: Multilocus sequence typing
nGBS: Noninvasive infection-related GBS isolates
NT: Nontypable serotype
PI: Pilus island
PCR: Polymerase chain reaction
ST: Sequence type

## References

[1] Edmond KM, Kortsalioudaki C, Scott S, Schrag SJ, Zaidi AK, Cousens S, Heath PT. Group B streptococcal disease in infants aged younger than 3 months: systematic review and meta-analysis. Lancet. 2012;379(9815):547–56. 22226047 10.1016/S0140-6736(11)61651-6

[2] Raabe VN, Shane AL. Group B Streptococcus (Streptococcus agalactiae). Microbiol Spectr 2019, 7(2). 10.1128/microbiolspec.gpp3-0007-2018 PMC6432937 30900541

[3] Song SH, Lee HJ, Song ES, Ahn JG, Park SE, Lee T, Cho HK, Lee J, Kim YJ, Jo DS, et al. Changes in etiology of invasive bacterial infections in infants under 3 months of age in Korea, 2006–2020. Pediatr Infect Dis J. 2022;41(12):941–6. 36375095 10.1097/INF.0000000000003714

[4] Bliss SJ, Manning SD, Tallman P, Baker CJ, Pearlman MD, Marrs CF, Foxman B. Group B Streptococcus colonization in male and nonpregnant female university students: a cross-sectional prevalence study. Clin Infect Dis. 2002;34(2):184–90. 11740706 10.1086/338258

[5] Russell NJ, Seale AC, O’Driscoll M, O’Sullivan C, Bianchi-Jassir F, Gonzalez-Guarin J, Lawn JE, Baker CJ, Bartlett L, Cutland C, et al. Maternal colonization with Group B Streptococcus and serotype distribution Worldwide: systematic review and Meta-analyses. Clin Infect Dis. 2017;65(suppl2):S100–11. 29117327 10.1093/cid/cix658 PMC5848259

[6] Seale AC, Bianchi-Jassir F, Russell NJ, Kohli-Lynch M, Tann CJ, Hall J, Madrid L, Blencowe H, Cousens S, Baker CJ, et al. Estimates of the Burden of Group B Streptococcal Disease Worldwide for pregnant women, Stillbirths, and children. Clin Infect Dis. 2017;65(suppl2):S200–19. 29117332 10.1093/cid/cix664 PMC5849940

[7] Ballard MS, Schønheyder HC, Knudsen JD, Lyytikäinen O, Dryden M, Kennedy KJ, Valiquette L, Pinholt M, Jacobsson G, Laupland KB. The changing epidemiology of group B streptococcus bloodstream infection: a multi-national population-based assessment. Infect Dis (Lond). 2016;48(5):386–91. 26759190 10.3109/23744235.2015.1131330

[8] Lamagni TL, Keshishian C, Efstratiou A, Guy R, Henderson KL, Broughton K, Sheridan E. Emerging trends in the epidemiology of invasive group B streptococcal disease in England and Wales, 1991–2010. Clin Infect Dis. 2013;57(5):682–8. 23845950 10.1093/cid/cit337

[9] Teatero S, McGeer A, Li A, Gomes J, Seah C, Demczuk W, Martin I, Wasserscheid J, Dewar K, Melano RG, et al. Population structure and antimicrobial resistance of invasive serotype IV group B Streptococcus, Toronto, Ontario, Canada. Emerg Infect Dis. 2015;21(4):585–91. 25811284 10.3201/eid2014.140759 PMC4378482

[10] Björnsdóttir ES, Martins ER, Erlendsdóttir H, Haraldsson G, Melo-Cristino J, Kristinsson KG, Ramirez M. Changing epidemiology of group B streptococcal infections among adults in Iceland: 1975–2014. Clin Microbiol Infect. 2016;22(4):e379379–379316. 10.1016/j.cmi.2015.11.020 26691681

[11] Francois Watkins LK, McGee L, Schrag SJ, Beall B, Jain JH, Pondo T, Farley MM, Harrison LH, Zansky SM, Baumbach J, et al. Epidemiology of Invasive Group B Streptococcal infections among nonpregnant adults in the United States, 2008–2016. JAMA Intern Med. 2019;179(4):479–88. 30776079 10.1001/jamainternmed.2018.7269 PMC6450309

[12] Quincer EM, Cranmer LM, Kamidani S. Prenatal maternal immunization for Infant Protection: a review of the vaccines recommended, infant immunity and future research directions. Pathogens 2024, 13(3). 10.3390/pathogens13030200 PMC10975994 38535543

[13] Carreras-Abad C, Ramkhelawon L, Heath PT, Le Doare K. A vaccine against Group B Streptococcus: recent advances. Infect Drug Resist. 2020;13:1263–72. 32425562 10.2147/IDR.S203454 PMC7196769

[14] Imperi M, Pataracchia M, Alfarone G, Baldassarri L, Orefici G, Creti R. A multiplex PCR assay for the direct identification of the capsular type (Ia to IX) of Streptococcus agalactiae. J Microbiol Methods. 2010;80(2):212–4. 19958797 10.1016/j.mimet.2009.11.010

[15] Kannika K, Pisuttharachai D, Srisapoome P, Wongtavatchai J, Kondo H, Hirono I, Unajak S, Areechon N. Molecular serotyping, virulence gene profiling and pathogenicity of Streptococcus agalactiae isolated from tilapia farms in Thailand by multiplex PCR. J Appl Microbiol. 2017;122(6):1497–507. 28295891 10.1111/jam.13447

[16] Huebner EM, Gudjónsdóttir MJ, Dacanay MB, Nguyen S, Brokaw A, Sharma K, Elfvin A, Hentz E, Rivera YR, Burd N, et al. Virulence, phenotype and genotype characteristics of invasive group B Streptococcus isolates obtained from Swedish pregnant women and neonates. Ann Clin Microbiol Antimicrob. 2022;21(1):43. 36229877 10.1186/s12941-022-00534-2 PMC9560721

[17] Slotved HC, Møller JK, Khalil MR, Nielsen SY. The serotype distribution of Streptococcus agalactiae (GBS) carriage isolates among pregnant women having risk factors for early-onset GBS disease: a comparative study with GBS causing invasive infections during the same period in Denmark. BMC Infect Dis. 2021;21(1):1129. 34724923 10.1186/s12879-021-06820-2 PMC8561911

[18] Choi SJ, Kang J, Uh Y. Recent epidemiological changes in Group B Streptococcus among pregnant Korean women. Ann Lab Med. 2021;41(4):380–5. 33536356 10.3343/alm.2021.41.4.380 PMC7884197

[19] Yoon IA, Jo DS, Cho EY, Choi EH, Lee HJ, Lee H. Clinical significance of serotype V among infants with invasive group B streptococcal infections in South Korea. Int J Infect Dis. 2015;38:136–40. 26026823 10.1016/j.ijid.2015.05.017

[20] Kang HM, Lee HJ, Lee H, Jo DS, Lee HS, Kim TS, Shin JH, Yun KW, Lee B, Choi EH. Genotype characterization of Group B Streptococcus isolated from infants with Invasive diseases in South Korea. Pediatr Infect Dis J. 2017;36(10):e242–7. 28060047 10.1097/INF.0000000000001531

[21] Madrid L, Seale AC, Kohli-Lynch M, Edmond KM, Lawn JE, Heath PT, Madhi SA, Baker CJ, Bartlett L, Cutland C, et al. Infant Group B Streptococcal Disease Incidence and Serotypes Worldwide: systematic review and Meta-analyses. Clin Infect Dis. 2017;65(suppl2):S160–72. 29117326 10.1093/cid/cix656 PMC5850457

[22] Ji W, Liu H, Madhi SA, Cunnington M, Zhang Z, Dangor Z, Zhou H, Mu X, Jin Z, Wang A, et al. Clinical and molecular epidemiology of Invasive Group B Streptococcus Disease among infants, China. Emerg Infect Dis. 2019;25(11):2021–30. 31600132 10.3201/eid2511.181647 PMC6810193

[23] Margarit I, Rinaudo CD, Galeotti CL, Maione D, Ghezzo C, Buttazzoni E, Rosini R, Runci Y, Mora M, Buccato S, et al. Preventing bacterial infections with pilus-based vaccines: the group B streptococcus paradigm. J Infect Dis. 2009;199(1):108–15. 19086816 10.1086/595564

[24] Lu B, Wu J, Chen X, Gao C, Yang J, Li Y, Wang J, Zeng J, Fang Y, Wang D, et al. Microbiological and clinical characteristics of Group B Streptococcus isolates causing materno-neonatal infections: high prevalence of CC17/PI-1 and PI-2b sublineage in neonatal infections. J Med Microbiol. 2018;67(11):1551–9. 30265233 10.1099/jmm.0.000849

[25] Tsai IA, Su Y, Wang YH, Chu C. Alterations in genes rib, scpB and Pilus Island decrease the prevalence of predominant serotype V, not III and VI, of Streptococcus agalactiae from 2008 to 2012. Pathogens 2022, 11(10). 10.3390/pathogens11101145 PMC9611264 36297202

[26] Zhang L, Ma L, Zhu L, Zhou XH, Xu LJ, Guo C, Meng JH, Zhang XH, Liu QH, Huang R. Molecular characterization of pathogenic group B streptococcus from a tertiary hospital in Shanxi, China: high incidence of sequence type 10 strains in infants/pregnant women. J Microbiol Immunol Infect. 2021;54(6):1094–100. 32826191 10.1016/j.jmii.2020.07.018

[27] Springman AC, Lacher DW, Waymire EA, Wengert SL, Singh P, Zadoks RN, Davies HD, Manning SD. Pilus distribution among lineages of group b streptococcus: an evolutionary and clinical perspective. BMC Microbiol. 2014;14:159. 24943359 10.1186/1471-2180-14-159 PMC4074840

[28] Lazzarin M, Mu R, Fabbrini M, Ghezzo C, Rinaudo CD, Doran KS, Margarit I. Contribution of pilus type 2b to invasive disease caused by a Streptococcus agalactiae ST-17 strain. BMC Microbiol. 2017;17(1):148. 28673237 10.1186/s12866-017-1057-8 PMC5496222

[29] Liu Y, Liu J. Group B Streptococcus: virulence factors and pathogenic mechanism. Microorganisms 2022, 10(12). 10.3390/microorganisms10122483 PMC9784991 36557736

[30] Al Safadi R, Mereghetti L, Salloum M, Lartigue MF, Virlogeux-Payant I, Quentin R, Rosenau A. Two-component system RgfA/C activates the fbsB gene encoding major fibrinogen-binding protein in highly virulent CC17 clone group B Streptococcus. PLoS ONE. 2011;6(2):e14658. 21326613 10.1371/journal.pone.0014658 PMC3033900

[31] Delara M, Vadlamudi NK, Sadarangani M. Strategies to prevent early and Late-Onset Group B Streptococcal Infection via interventions in pregnancy. Pathogens 2023, 12(2). 10.3390/pathogens12020229 PMC9959229 36839501

[32] Heath PT. Status of vaccine research and development of vaccines for GBS. Vaccine. 2016;34(26):2876–9. 26988258 10.1016/j.vaccine.2015.12.072

[33] Martins ER, Andreu A, Correia P, Juncosa T, Bosch J, Ramirez M, Melo-Cristino J. Group B Streptococci causing neonatal infections in barcelona are a stable clonal population: 18-year surveillance. J Clin Microbiol. 2011;49(8):2911–8. 21697333 10.1128/JCM.00271-11 PMC3147731

